# Factors Associated with Length of Hospital Stay among COVID-19 Patients in Saudi Arabia: A Retrospective Study during the First Pandemic Wave

**DOI:** 10.3390/healthcare10071201

**Published:** 2022-06-27

**Authors:** Abdullah K. Alahmari, Ziyad S. Almalki, Ahmed A. Albassam, Mohammed M. Alsultan, Ahmed M. Alshehri, Nehad J. Ahmed, Abdulhadi M. Alqahtani

**Affiliations:** 1 Department of Clinical Pharmacy, College of Pharmacy, Prince Sattam Bin Abdulaziz University, Al-Kharj 11942, Saudi Arabia; z.almalki@psau.edu.sa (Z.S.A.); aa.albassam@psau.edu.sa (A.A.A.); ah.alshehri@psau.edu.sa (A.M.A.); n.ahmed@psau.edu.sa (N.J.A.); 2 Department of Pharmacy Practice, College of Clinical Pharmacy, Imam Abdulrahman Bin Faisal University, Dammam 34212, Saudi Arabia; mmaalsultan@iau.edu.sa; 3 Clinical Research Department, Research Center, King Fahad Medical City, Riyadh 12231, Saudi Arabia; ph.abdalhadi@gmail.com

**Keywords:** COVID-19, hospital stay, hospitalisation, health systems

## Abstract

The COVID-19 pandemic severely affected healthcare systems and tested their preparedness. To date, the length of hospital stay (LoHS) and its factors among COVID-19 patients has not been thoroughly studied. Moreover, it is essential to identify the features of these patients. Adult COVID-19 patients in Saudi Arabia with complete electronic medical records and who were hospitalised for >1 day between 1 May 2020 and 30 July 2020 at one of two hospitals were considered for this retrospective cohort study. Descriptive statistics and multivariate generalized linear models were performed using the data. Of the patients, 34% were ≥50 years old and 80.14% were female. More than 70% had mild-to-moderate symptoms; 45% had either diabetes or hypertension. The median LoHS was 7.00 days (IQR: 3–11). Patients who were females, had either critical or severe disease, were on mechanical ventilation, had diabetes, and administered ceftriaxone had significantly longer LoHS (*p* < 0.05). Patients administered zinc sulphate had significantly shorter LoHS (*p* = 0.0008). During the first pandemic wave, COVID-19 patients were hospitalised for 7 days. Healthcare professionals should pay more attention to women, patients with diabetes, and those with severe or critical symptoms. Unnecessary use of ceftriaxone should be minimised, and zinc sulphate can be administered.

## 1. Introduction

Coronavirus disease (COVID-19) is caused by the severe acute respiratory syndrome coronavirus 2 (SARS-CoV-2) that originated in Wuhan city in China in December 2019. Since then, it has spread worldwide and has caused millions of infections and deaths [1,2]. Although there are a high number of confirmed COVID-19 cases, the fatality rate of COVID-19 is lower than that of other previous outbreaks, such as the Middle East respiratory syndrome coronavirus (MERS CoV) and severe acute respiratory syndrome coronavirus (SARS-CoV) [1,3,4]. In Saudi Arabia, the first COVID-19 confirmed case was reported on 2 March 2020 [5].

The manifestations of COVID-19 vary from mild symptoms in some patients to severe symptoms in others [1,6]. Mild symptoms include dyspnoea, fever, anorexia, cough, sore throat, and fatigue [7,8]. In some patients, the virus may cause severe complications, such as sepsis, neurological symptoms, pneumonia, hyperinflammation, acute respiratory distress syndrome, or multi-system organ failure [5,9].

Predicting the incidence of COVID-19 and estimating the total length of hospital stay (LoHS) in patients are necessary to understand the impact of COVID-19 on hospital capacity [10]. It is also important to ensure that hospitals can provide adequate bed capacity without unnecessarily restricting care for other patients [10]. Rees et al. found that the LoHS within China ranged from 4 to 53 days, and that outside China ranged was from 4 to 21 days [11]. Phua et al. reported that LoHS varied significantly among countries even before the pandemic [12]. Alwafi et al. reported that the median LoHS among COVID-19 patients was six days (range LoHS 0–55 days) in Saudi Arabia [13]. Further, Alsofayan and colleagues reported that the rate of hospitalisation among all confirmed COVID-19 cases in March 2020 was 71.6% in Saudi Arabia, with a 0.65% mortality rate [14]. According to Alghamdi, the LoHS among COVID-19 patients in Saudi Arabia ranged from 4 to 15.6 days [15].

The LoHS for COVID-19 depends on the clinical situation of the patients, the local guidelines, and the capacity of hospitals [16,17]. The demand for inpatient facilities is increasing with the number of COVID-19 cases [18]. Alwafi et al. reported that, among the hospitalised COVID-19 patients in Saudi Arabia, venous thromboembolism and radiological evidence of pneumonia, age, higher D-dimer values, and chronic comorbidities are the major risk factors associated with an increased LoHS and a higher risk of death [13].

As the rate of hospitalisation among COVID-19 patients in Saudi Arabia at the beginning of epidemic was high, it was necessary to identify different factors of longer LoHS. Prioritising patients, contingency planning, and quick decision-making processes are also important [11]. Therefore, the aim of the present study was to identify the factors of the length of hospital stay for COVID-19 patients in two referral hospitals in Saudi Arabia.

## 2. Methods

### 2.1. Study Design/Setting

A retrospective cohort study was conducted using electronic medical records (EMRs) of hospitalised COVID-19 patients between 1 May 2020 and 30 July 2020. This study took place at King Fahad Medical City (KFMC) and Prince Mohammed Bin Abdulaziz Hospital (PMAH). Both KFMC and PMAH were among the major referral hospitals located in eastern Riyadh to receive COVID-19 patients at the start of the coronavirus epidemic in the region.

### 2.2. Study Population

The study included male and female adults of all ages who suffered from COVID-19, confirmed using real-time reverse transcriptase-polymerase chain reaction (RT-PCR). Only patients having complete EMRs and a LoHS of more than one day were included in our study.

### 2.3. Data Collection

The medical records of all the hospitalised patients were retrieved. Data were collected from COVID-19 positive patients by trained medical personnel. A well-designed and organised checklist was used to obtain information from patients’ medical records, which consists of basic information (age, sex, smoking status, and nationality) in addition to data about clinical characteristics including all existing chronic conditions, laboratory data, treatment, and outcomes.

### 2.4. Dependent and Independent Variables

The LoHS in this study was defined as the number of days a patient spent in the hospital which was calculated using patient EMR by subtracting the admission date from the discharge date.

Independent variables included patient age; sex; nationality; body mass index (BMI); severity of symptoms (a patient was classified as mild-to-moderate if he/she had a fever “up to 39.4 °C”, fatigue, cough, sore throat, headache, and nasal congestion; patients who experienced high fever “above 39.4 °C”, shortness of breath, severe chest pain, and severe muscles pain were classified as severe cases; and patients who required mechanical ventilation and/or ICU admission were classified as critical cases); status of physical examination; status of X-ray examination; status of mechanical ventilation; symptoms of the disease (such as vomiting, fever, dry cough, nausea, diarrhoea, abdominal pain, sore throat, and dyspnoea); comorbidities, identified based on whether the patient had a confirmed diagnosis with any of the following conditions: diabetes (if the patient’s EMR has a confirmed diagnosis of diabetes by a medical professional and/or if the patient is currently using antihyperglycemic medications along with an HbA1c level ≥ 7%), cardiovascular disease (CVD) (if the patient has any confirmed diagnosis of angina, myocardial infarction, stroke, or heart failure), asthma (if the patient has a confirmed diagnosis with asthma and/or currently administering asthma medications), hypertension (if the patient’s EMR has a confirmed diagnosis with hypertension by a medical professional and/or if the patient is currently using antihypertensive medications), cancer (when a patient has had a confirmed diagnosis of any type of cancer and/or currently on any type of anticancer therapy, and kidney disease (if the patient had a urinary albumin creatinine ratio ≥ 30 mg/g and/or estimated glomerular filtration rate < 60 mL/min/1.73 m^2^); and treatment administered (e.g., enoxaparin, doxycycline, vancomycin, ceftriaxone, favipiravir, paracetamol, zinc sulphate, and piperacillin/tazobactam).

### 2.5. Statistical Data Analysis

Descriptive statistics such as mean ± standard deviation (SD) and/or median for continuous variables and frequency for categorical variables were used to summarise the baseline characteristics of the study population.

To calculate the average LoHS, we calculated the total number of days a patient stayed in the hospital for all patients and divided it by the total number of patients in this study. To identify the factors of LoHS a univariate generalized linear regression was carried out for each independent variable separately, and independent variables with *p* > 0.1 were excluded from the multivariate model. Then, the identified factors were fitted into a multivariate generalized linear model (GLM) with a least square mean statement to test the association between these factors and LoHS. We considered *p* < 0.05 as statistically significant. All analyses were performed using SAS software (version 9.4) and Microsoft Excel.

## 3. Results

A combined total of 977 COVID-19 positive patients were admitted to the studied hospitals between 1 May 2020 and 30 July 2020 and were chosen for this study. Nearly 34% of the patients were ≥50 years of age and 80.14% were female (Table 1). Additionally, 70% of the included patients were citizens of Saudi Arabia, and more than 60% of them were either overweight or obese. Regarding the severity of symptoms upon hospitalisation, we found that approximately 70% of the patients had mild to moderate symptoms, nearly 18% of them were hospitalised with severe symptoms, and 12% of them had critical symptoms. Furthermore, more than 50% of the patients had normal physical examinations, 60% had normal chest X-rays, and approximately 14% of them needed mechanical ventilation. Hypertension and diabetes were the most common comorbidities (24% and 23%, respectively), and the most common symptoms among the patients were fever, dry cough, and dyspnoea (78%, 72%, and 38%, respectively). Moreover, regarding the potential treatments of the admitted patients, 36% of the patients had received ceftriaxone, 27% received enoxaparin, 23% received paracetamol, 13.31% received zinc sulphate, and 9.11% received piperacillin/tazobactam. The baseline characteristics of the study participants are shown in Table 1.

Table 2 shows the average LoHS and its independent factors due to coronavirus infection. A total of 8581 days of hospitalization (range 1–70 days) were utilised by our sample of 977 patients which indicates a median LoHS of 7.0 days (IQR = 3–11). A comparison of the factors revealed that female patients were associated with significantly longer LoHS than male patients (estimate = 2.39 days, *p* = 0.0002). In addition, patients who had either severe or critical symptoms were associated with longer LoHS than those with mild-to-moderate symptoms (estimates = 3.19 days, *p* < 0.0001 and 6.19 days, *p* < 0.0001, respectively). The results also showed that patients who were admitted with abnormal chest X-rays were associated with significantly longer LoHS (2.26 days, *p* = 0.0018) than patients with normal chest X-rays. Similarly, patients who were on mechanical ventilation, had diabetes, or had kidney disease were associated with longer LoHS than those without these conditions (4.61 days, *p* = 0.0005; 1.46 days, *p* = 0.0233; and 4.69 days, *p* = 0.0167, respectively). Interestingly, we found that patients who received ceftriaxone had longer LoHS (estimate = 2.76 days, *p* < 0.0001) while those who received zinc sulphate were associated with significantly shorter LoHS (estimate = −2.76 days, *p* = 0.0008).

## 4. Discussion

Nearly 34% of the included patients were ≥50 years of age; most of them were females and more than half of them were either overweight or obese. Verity et al. reported that old age is a risk factor for the severity of coronavirus disease compared to young and middle-aged individuals due to their low immunity and higher prevalence of chronic illnesses [19]. Centers for Disease Control and Prevention reported that older adults are at the highest risk of getting very sick from COVID-19, and that more than 81% of COVID-19 deaths occur in people over age 65 [20]. Dowd et al. and Caramelo et al. reported that gender is one of the factors of heterogeneity in COVID19 mortality and that men have a higher risk than women [21,22]. Contradictory to our results, Jin et al. reported that, while males and females have the same prevalence, males with COVID-19 are more at risk of death and have worse outcomes than females [23]. Moreover, Abate et al. found that the prevalence of symptomatic COVID-19 was higher in males than in females [24].

Our results indicated that the median LoHS among COVID-19 patients was 7.00 days (IQR, 3–11). However, a previous study conducted by Alwafi et al. [13] reported slightly different findings (median LoHS was 6.0 days) among COVID-19 patients in Saudi Arabia. Several reasons could explain the inconstancy between these findings. First, the median LoHS obtained from our study was slightly longer than that reported by Alwafi et al., possibly due to the fact that the range of hospital stays among our patients was in fact more right-skewed compared to theirs (1–70 days vs. 0–55 days). Such a difference could prolong the median obtained from our study. Second, unlike Alwafi et al.’s study, our data had no patients with 0 days LoHS which might explain why our median was slightly higher.

The most common comorbidities in our study participants were hypertension and diabetes, and the most common symptoms were fever, dry cough, and dyspnoea. Killerby et al. and Tenforde et al. stated that symptoms of COVID-19 may differ with the severity of the disease, and that dyspnoea is more frequently reported among people who are hospitalised with COVID-19 than among non-hospitalised patients [25,26]. Cai et al. and Dong et al. reported that atypical presentations of COVID-19 occur in older adults and in people with medical comorbidities who may experience more respiratory symptoms and fever than those who are younger or do not have comorbidities [27,28]. Moreover, Abougazia et al. reported that patients with diabetes, cardiac disease, hypertension, obesity, and chronic kidney disease were at a higher risk of positive chest X-ray findings [29]. Alguwaihes et al. found that, among the 439 COVID-19 patients who were included in their study, the most prevalent comorbidity was vitamin D deficiency (74.7%), followed by diabetes (68.3%), hypertension (42.6%), and obesity (42.2%) [3]. Similarly, diabetes was the most prevalent comorbid condition among our patients, followed by hypertension, and 66.64% were either overweight or obese.

The most commonly used medications in our study were ceftriaxone, enoxaparin, paracetamol, zinc sulphate, and piperacillin/tazobactam. Several studies have shown that several patients require antibiotics and anticoagulants. Adebisi et al. reported that various antibiotics, such as azithromycin, ceftriaxone, amoxicillin-clavulanic acid, and piperacillin/tazobactam, were recommended for use in the treatment of COVID-19 [30]. Chedid observed in a review that half of the included studies reported the occurrence of a bacterial co-infection or complication and that the pooled data of studies showed half of the patients receiving antibiotics were neither critical nor severe [31]. In contrast, Ceftriaxone was administered to only 36.34% of our sample. Such a lower percentage could be due to the fact that nearly 70% of our patients had mild-to-moderate disease severity. Tang et al. found that among 449 severe COVID-19 patients, enoxaparin use appeared to improve survival when compared with no pharmacologic prophylaxis, particularly in patients with high D-dimer levels [32]. Moreover, Billett et al. observed that COVID-19 patients with moderate or severe illness benefit from the use of anticoagulants and that enoxaparin is effective in decreasing mortality [33]. Several studies have also recommended the use of paracetamol and zinc supplements for the treatment of COVID-19. The Saudi Ministry of Health reported that in suspicious cases paracetamol is the preferred agent for treating fever and pain [34]. Leal et al. suggested paracetamol as an alternative to ibuprofen for treating COVID-19 symptoms [35]. Yasui et al. reported that the available information suggests that zinc deficiency is related to increased severity of the COVID-19 [36]. Furthermore, Wessels et al. and Skalny et al. reported many published data on the potential use of zinc as a therapeutic agent against COVID-19 [37,38].

Our study showed that patients who experienced significantly extended LoHS were females, patients with either critical or severe symptoms, those with abnormal chest X-rays, those on mechanical ventilation, and those having diabetes and/or kidney disease. The study also showed that patients who received ceftriaxone had significantly longer LoHS, but those who received zinc sulphate had significantly shorter LoHS. Similar to our findings, Alwafi et al. reported that hospitalized COVID-19 patients with end-stage renal diseases had a significantly higher risk of mortality and a significantly prolonged LoHS [13]. Mahboub et al. found that diagnosis at admission, painkiller usage, ventilator intubation, azithromycin usage, antiviral usage, anti-inflammatory agent usage, vitamin C usage, urea test results, platelet count, haemoglobin levels, dimer levels, and potassium levels were significant predictors of LoHS [39]. Moreover, Wang et al. reported that age and clinical grade were strongly related to the length of stay (*p* < 0.01) and that a longer LoHS was associated with ≥45 years of age, severe illness, and admission to a provincial hospital [40]. Our results of prolonged LoHS among patients with either severe or critical symptoms were aligned with Wang et al.’s findings. Álvarez-Esteban et al. found that the hospitalisation rate for those over 69 is 27.2% and for those under 70 is 5.3%; furthermore, the hospitalisation rate for males is 14.5% and for females 8.3% [41]. They also reported that, among patients with chronic diseases, the highest rates of hospitalisation were 26.3% for kidney disease and 26.1% for diabetes [41]. Furthermore, Guo et al. concluded that fever, female sex, higher creatinine levels, and chronic liver or kidney disease before admission were associated with prolonged LoHS in COVID-19 patients [42]. Alqassieh et al. reported that patients presenting with malaise or elevated WBC count along with smoking habits were associated with a shorter LoHS, but the loss of taste and chills or rigors at presentation was associated with a longer LoHS [43].

Our results indicated that COVID19 patients who had diabetes or kidney disease were associated with significantly longer LoHS compared to those without these comorbidities. It is very difficult to judge whether the cause of longer LoHS was due to the COVID-19 infection or the comorbid disease itself. However, Sanyaolu et al. conducted a systematic review of published papers between January and 20 April 2020 to examine the impact of comorbidities on COVID-19 disease progression and outcomes [44]. Sanyaolu et al. concluded that patients with comorbidities, especially hypertension and diabetes, were at higher risk of developing severe disease symptoms and progression compared to those without such comorbidities. Additionally, Singh et al. have conducted a meta-analysis of 29 studies published between 1 December 2019 and 20 August 2020 to examine the association between acute kidney injury (AKI) and chronic kidney disease (CKD) and COVID-19 disease severity [45]. Their findings stated that both AKI and CKD were associated with significantly higher risks of severe COVID-19 cases (OR: 8.28; 4.42–15.52; *p* < 0.00001, and 1.70; 95% CI: 1.21–2.40; *p* = 0.002, respectively). Based on the findings from these previous studies, we believe that patients with diabetes and/or kidney disease in our study were vulnerable to more severe COVID-19 symptoms, which lead to the prolonged LoHS observed among these patients.

COVID-19 patients in our study who received Ceftriaxone had significantly longer LoHS. Such finding is aligned with the fact that COVID-19 is a viral disease, and without a confirmed bacterial co-infection, antibiotics are not recommended. The World Health Organization (WHO) has clearly stated that, because COVID-19 is caused by a virus, antibiotics should not be used to prevent or treat it [46]. Additionally, COVID-19 could weaken the immune system of some patients, making them vulnerable to bacterial co-infection which may represent a reasonable indication for antibiotic prescription only by healthcare providers [46]. Despite the fact that COVID-19 is caused by a virus, antibiotic prescriptions were prevalent, especially during the first pandemic wave of COVID-19. In a Chinese single-centre retrospective study between 1–20 January 2020, Chen et al. reported that, despite a proven bacterial co-infection rate of only 1%, antibiotics were administered to 71% of COVID-19 patients in their centre [47]. Moreover, Zhou et al. reported that 95% of COVID-19 patients had been placed on antibiotic regimens, even though a secondary bacterial infection was only found in 15% of the patients [8]. While bacterial co-infection was infrequent among COVID-19 patients, Bendala Estrada et al. found that antibiotic use was high. They stated that there is inadequate evidence to justify empiric antibiotic usage for COVID-19 patients [48]. Additionally, Chitungo et al. stated that the COVID-19 pandemic has led to extensive use of antibiotics due to the lack of knowledge about the disease at the early stages of the pandemic. They concluded that such inappropriate use of antibiotics during COVID-19 has intensified the risk of antimicrobial resistance (AMR) [49].

There are some limitations to this study that need to be taken into account. First, the data analysed in this study was relatively old (May–July 2020) and our findings might not represent the current disease status. Results obtained from recent data would be different due to the introduction of COVID-19 vaccination and new anti-viral therapies that provide significant disease spread protection, minimized symptom severity, and reduced healthcare services utilization. Second, because this study only used data from two hospitals, extrapolating its findings to the entire kingdom is difficult. Third, the data was gathered at the beginning of the pandemic, and it is possible that some psychological and societal factors were among the causes of the prolonged stay observed among our patients. Additionally, there was a lack of vaccines and information about the disease at the time of this study, which may have contributed to the prolonged hospital stay. Finally, our study was conducted in two of Riyadh’s largest hospitals for corona cases, especially critical cases that may have a longer hospital stay due to their disease severity.

## 5. Conclusions

The study showed that patients who experienced significantly extended hospitalisation stays were predominantly women, patients with either critical or severe symptoms, those with abnormal chest X-rays, those on mechanical ventilation, those with diabetes, those diagnosed with kidney disease, or those who received ceftriaxone. Understanding the risk factors that increase the length of stay of COVID-19 patients is critical for managing the patients appropriately and allocating the available resources efficiently.

## Figures and Tables

**Table 1:** Baseline Characteristics of the Study Patients (*n* = 977).

Characteristics	*n* (%)
Age (years)	
18–34	276 (28.25)
35–49	368 (37.67)
50–64	229 (23.44)
≥65	104 (10.64)
Sex	
Male	194 (19.86)
Female	783 (80.14)
Nationality	
Saudi Arabian	687 (70.32)
Non-Saudi Arabian	290 (29.68)
Body Mass Index	
Normal Weight	326 (33.37)
Overweight	359 (36.75)
Obese	292 (29.89)
The severity of Presenting Disease	
Mild to Moderate	680 (69.60)
Severe	177 (18.12)
Critical	120 (12.28)
Does the Patient have Symptoms	900 (92.12)
Normal Physical Examination	510 (52.20)
Normal Chest X-ray	594 (60.80)
Patient on Mechanical Ventilation	130 (13.31)
Plan of Treatment	
Discharge	126 (12.90)
ICU	183 (18.73)
Isolation	668 (68.37)
Comorbidities	
Cardiovascular Diseases	36 (3.68)
Hypertension	220 (22.52)
Diabetes	231 (23.64)
Kidney Diseases	17 (1.74)
Asthma	28 (2.87)
Cancer	11 (1.13)
Mental Disorders	16 (1.64)
Symptoms of Presenting Complain	
Fever	757 (77.48)
Nausea	33 (3.38)
Vomiting	77 (7.88)
Dry Cough	704 (72.06)
Dyspnoea	373 (38.18)
Fatigue	44 (4.50)
Abdominal Pain	33 (3.38)
Diarrhoea	97 (9.93)
Myalgia	24 (2.46)
Sore Throat	57 (5.83)
Treatment of Current Complain	
Doxycycline	57 (5.83)
Enoxaparin	260 (26.61)
Favipiravir	45 (4.61)
Paracetamol	222 (22.72)
Piperacillin/Tazobactam	89 (9.11)
Vancomycin	28 (2.87)
Ceftriaxone	355 (36.34)
Zinc Sulphate	130 (13.31)

**Table 2:** Average length of hospital stay and the independent factors associated with the length of hospital stay obtained from the generalized linear model (GLM).

Parameter	Length of Stay		GLM Estimates		
Total Days of Hospitalisation	Mean	Standard Error	Estimate	Standard Error	*p* Value
8581	median 7.00	(IQR, 3–11)			
Intercept			4.41	0.36	<0.0001
Sex					
Female	10.70	0.57	2.39	0.64	0.0002
Male	8.31	0.28	Reference		
The severity of presenting disease					
Severe	10.63	0.68	3.19	0.82	<0.0001
Critical	13.64	1.28	6.19	1.49	<0.0001
Mild to Moderate	7.45	0.37	Reference		
Chest X-ray examination					
Abnormal	10.16	0.51	2.26	0.72	0.0018
Normal	7.90	0.38	Reference		
Is the patient on mechanical ventilation?					
Yes	12.78	1.17	4.61	1.32	0.0005
No	8.17	0.31	Reference		
Does the patient have diabetes?					
Yes	9.90	0.55	1.46	0.64	0.0233
No	8.44	0.29	Reference		
Does the patient have kidney disease?					
Yes	13.39	1.94	4.69	1.95	0.0167
No	8.70	0.25	Reference		
Has the patient received ceftriaxone?					
Yes	10.54	0.44	2.76	0.58	<0.0001
No	7.78	0.33	Reference		
Has the patient received zinc sulphate?					
Yes	6.39	0.76	(2.76)	0.82	0.0008
No	9.15	0.27	Reference		

## Data Availability

Not applicable.
